# Address to the Medical Profession

**Published:** 1846-12

**Authors:** J. Knight

**Affiliations:** Chairman


*Address to the Medical Profession, in relation to the objects of the National Medical Association, by the Committee appointed for that purpose.*

Abstract from the Minutes of the Proceedings of the National Medical Convention, held in the City of New York, in the month of May, 1846—

*Resolved,* That a committee of seven be appointed to prepare and issue an address to the different regularly organized medical societies, and chartered medical schools of the United States, setting forth the objects of the National Medical Association, and inviting them to send delegates to a Convention to be held in Philadelphia, on the first Wednesday in May, 1847.

In obedience to the above resolution, the committee appointed for the purpose present the following remarks to the members of the medical profession generally, as well as to the several bodies named in the resolution:—

In compliance with an invitation from the Medical Society of the State of New York, delegates from various medical societies and schools met in Convention in the city of New York, on the first Tuesday of May, 1846. About one hundred members were present, representing thirty-four different medical associations, and residents in sixteen different states of the Union. The object of the Convention was, by a concert of action of medical men from every part of the country, to advance the interests, the honor, and the usefulness of the profession. The result of their deliberations, as shown in their published proceedings, has been widely circulated, so that all who take an interest in such matters, are probably fully informed of it; still, some remarks upon the course which was pursued, together with the reasons for it, may be appropriate.

The first object which engaged the attention of the Convention was the necessity of forming some plan of organization, by which medical men in every part of the country may communicate with each other, and act in concert, in regard to their common interests. The general opinion was, that this desirable object would be best attained by the formation of a National Medical Association, to consist of members from every part of the country, who should meet together for consultation and action, at such times and places as they might deem expedient. Although no doubt was expressed of the propriety of such an organization, there were other subjects connected with it, such as, who should be members of the association? how and by whom should they be selected? if delegates, what should be their number? what power should be conferred upon them, and how far should their acts be binding upon the bodies whom they represent? concerning which there would probably be difference of opinion. As no one had been charged with the consideration of these subjects, no definite plan of organization had been prepared, upon which the Convention could act with that careful deliberation which the importance of the business demanded. It was thought best, therefore, to postpone all definite action upon it until a future Convention. This opinion was strengthened by the fact, that although the number of members present was larger than had been anticipated, yet many most respectable medical societies and schools, who are doubtless equally solicitous with those who were present, for the welfare of the profession, and as ready to promote its interests, were not represented. This deficiency, it was hoped, would be remedied in a future Convention. In the meantime, the consideration of these subjects was intrusted to a committee, who will prepare a plan for a National Medical Association, which will be presented to the proposed future Convention.

The other subjects which engaged the attention of the Convention, related principally to medical education; such as the qualifications which should be required of those about to engage in the study of medicine; the course of study which should be pursued by them; the mode of examination which should be adopted, and others of a similar kind. For reasons like those given above, these several matters were also placed in the hands of committees to examine, and report upon them. The same course was also pursued in regard to the preparation of a code of medical ethics, by which the intercourse of physicians with each other, and with the public, shall be regulated.

From this statement it will be seen that matters of great interest are presented for consideration, and that if wise measures in regard to them are adopted, such errors and abuses as may be found to exist, will be corrected; the profession will be guarded against the admission of incompetent or unworthy members; the purity, professional and moral, of those who are allowed to continue in it, will be preserved; and that thus full assurance, such as it has a right to demand, will be given to the community, that all who are acknowledged by medical men to be of their number, are worthy and competent to perform the duties, and to sustain the resposibilities of an arduous and honorable profession.

It remains to be seen whether these desirable objects shall be accomplished. It is obvious that this can be done only by general and united exertion. All partial or divided action will be unavailing. It is equally true, that whatever is done, must be accomplished by medical men themselves. In some countries, the interests of the medical profession, like others of great importance to the community, are regulated, sustained, and protected by public law. In this country, no general law, even if it were desirable, can ever exist. In many of the states, no laws upon this subject have ever been enacted; in several, where they have formerly existed, they have been repealed, and in those where they still remain, there is a general complaint of their inefficiency. In this state of things, the only resource which remains, is, for medical men to establish and enforce among themselves such regulations as shall purify and elevate their own body, and thus more fully command the respect and confidence of their fellow-men. The proposed association, if it becomes general, may be the means of accomplishing much of this good work. The opinions of such a body of the most respectable membeıs of the profession, enjoying the confidence of their brethren, and of the public, freely expressed after full consultation and careful deliberation, although not clothed with the authority of the law, will still command respect, and for the most part, compliance. A public opinion in regard to the subjects decided upon, will be created, which will be more controlling than law. It is by creating and sustaining a sound and healthy public opinion, that the association will prove most beneficial.

In calling the attention of the physicians of this country to such an effort at this time, it is not intended to express the opinion, for no such opinion is entertained, that they are behind those of other countries, or the members of the other professions in this, in general intelligence, in scientific attainments, or in practical skill. Still it is to be remembered that the present is peculiarly an age of advance in every department of science, and that at such a time to rest satisfied with present attainments, and to make no provisions for increased acquisitions, is practical retrocession.

In this state of things, the committee feel themselves fully authorized to call upon the medical profession throughout the country to consider, carefully and deliberately, the matters which have been presented to them, and upon which a future convention will be called on to act. They also earnestly invite all medical societies, the faculties of all medical colleges, and all similar associations to appoint delegates to meet in Convention in Philadelphia, on the first Wednesday in May, 1847.

It is confidently believed that a Convention thus constituted, embodying the wisdom, and acting under the sanction, and with the authority of the united profession, will devise such measures as shall command the respect of all who are interested in the promotion of the medical science, and the physical welfare of man.

In behalf of the Committee,

J. KNIGHT, M.D., *Chairman.*

